# Sweet-like syndrome secondary to chimeric antigen receptor T-cell therapy for multiple myeloma: a case report

**DOI:** 10.3389/fimmu.2026.1844119

**Published:** 2026-05-28

**Authors:** Xianfeng Ouyang, Yan Huang, Yanfang Huang, Si Yi, Yulu Wang, Linchun Fang

**Affiliations:** 1 Department of Hematology, Jiujiang First People’s Hospital, Jiujiang, Jiangxi, China; 2 Department of Respiratory and Critical Care Medicine, The First Affiliated Hospital, Jiangxi Medical College, Nanchang University, Nanchang, China; 3 Department of Hematology, The First Affiliated Hospital, Jiangxi Medical College, Nanchang University, Nanchang, China

**Keywords:** BCMA, chimeric antigen receptor T-cell therapy, cutaneous toxicity, multiple myeloma, sweet’s syndrome

## Abstract

**Background:**

Chimeric antigen receptor T-cell therapy targeting B-cell maturation antigen (BCMA CAR-T) has significantly improved outcomes in relapsed/refractory multiple myeloma (R/RMM), yet its spectrum of immune-related adverse events continues to be defined. Among these, cutaneous toxicities are not unusual, but frequently receive insufficient attention. Delayed-onset cutaneous reactions, in particular, may be underreported.

**Case report:**

We report a patient with R/RMM who achieved stringent complete response (sCR) following BCMA CAR-T therapy and developed delayed-onset cutaneous lesions on day 50 post-infusion. Histopathology revealed dense dermal neutrophilic infiltration, consistent with Sweet’s syndrome. However, the patient lacked typical clinical features such as fever, arthralgia, or peripheral neutrophilia, prompting a diagnosis of ‘Sweet-like syndrome’ associated with CAR-T therapy. The lesions resolved spontaneously without systemic glucocorticoid therapy.

**Conclusion:**

To our knowledge, virtually no cases of Sweet’s syndrome or Sweet−like syndrome have been reported previously after BCMA CAR−T therapy, broadening the recognized spectrum of CAR−T−related cutaneous adverse events and underscoring the importance of early biopsy for atypical, delayed−onset rashes—rather than empiric systemic corticosteroid treatment—to guide appropriate management.

## Introduction

Multiple myeloma (MM) remains an incurable clonal plasma cell malignancy, and despite the emergence of novel agents, most patients eventually experience relapse (1). Chimeric antigen receptor T-cell therapy targeting B-cell maturation antigen (BCMA CAR-T) has provided deep and durable responses for patients with relapsed/refractory multiple myeloma (R/RMM) (2). However, this revolutionary therapy is accompanied by distinctive immune-related adverse events, including cytokine release syndrome (CRS), immune effector cell-associated neurotoxicity syndrome (ICANS), immune effector cell-associated hematotoxicity (ICAHT), and infections, which have become the focus of clinical management (3). Cutaneous toxicities following CAR-T therapy are not uncommon but often receive insufficient attention. A review by Nusbaum et al. found that while clinical trial data reported skin toxicity rates ranging from 4% to 36%, only a small number of published case reports existed, and merely two originated from dermatology services. This discrepancy suggests that many cutaneous reactions are managed by oncologists without dermatology consultation or publication, resulting in systematic underrecognition (4). Delayed−onset reactions, which occur outside the acute monitoring period, are probably even more likely to go unreported. A large-scale database analysis revealed a significantly increased reporting rate of severe cutaneous reactions associated with CAR-T therapy, with both cutaneous and vascular adverse event groups correlating with high mortality (5). These findings underscore the clinical need for improved management strategies and mechanistic understanding of CAR-T-related skin toxicities.

Sweet syndrome has been reported as a skin disease closely associated with multiple myeloma itself, as well as its drug treatments (6–8). Sweet syndrome, also known as acute febrile neutrophilic dermatosis, is characterized by painful erythematous papules, plaques, or nodules, along with fever and peripheral neutrophilia. The histopathological hallmark is dense neutrophilic infiltration in the dermis, without evidence of vasculitis (9).

We report a case of a patient with R/RMM who developed Sweet-like syndrome following BCMA CAR-T therapy. To our knowledge, this is the first described case of Sweet-like syndrome occurring after BCMA−directed CAR−T therapy, expanding the recognized spectrum of cutaneous adverse events and informing clinical management.

## Case presentation

A 53-year-old male was diagnosed with IgD-λ multiple myeloma (Durie-Salmon stage II, ISS stage III). From October 2023 to April 2024, he received five cycles of VRd induction therapy (bortezomib, lenalidomide, dexamethasone). He subsequently discontinued treatment on his own. In December 2024, he presented with back pain and disease recurrence. He then received two additional cycles of VRd re-induction therapy; however, restaging confirmed progressive disease.

The patient then opted for academic BCMA CAR-T. Peripheral blood mononuclear cells were collected on April 16, 2025, for CAR-T manufacturing. Lymphodepleting chemotherapy with the FC regimen (cyclophosphamide 300 mg/m², fludarabine 50 mg/m2/day intravenously for three consecutive days) was initiated on May 3, 2025. BCMA CAR-T cells (2 × 10^6^/kg, 4-1BB costimulation) were infused on May 7 and May 8, 2025.

On day 4 post-infusion (May 11, 2025), the patient developed fever, with a peak temperature of 40 °C on May 13. Elevated cytokine levels were documented (IL-6 559.6 pg/mL, ferritin 1917 ng/mL, soluble IL-2 receptor 1103 U/mL). Extensive infectious workup, including blood next-generation sequencing, blood cultures, EBV-DNA, CMV-DNA, and respiratory viral antibody panels, was negative. Grade 1 CRS was diagnosed. The patient received prophylactic anti-infective therapy, fluid resuscitation, and a short course of dexamethasone, after which fever resolved.

On June 5, 2025, restaging bone marrow examination showed no evidence of clonal plasma cells, flow cytometry was negative for minimal residual disease, serum and urine immunofixation electrophoresis were negative, and free light chain ratios were normalized, confirming stringent complete remission (sCR).

**Figure 1:**
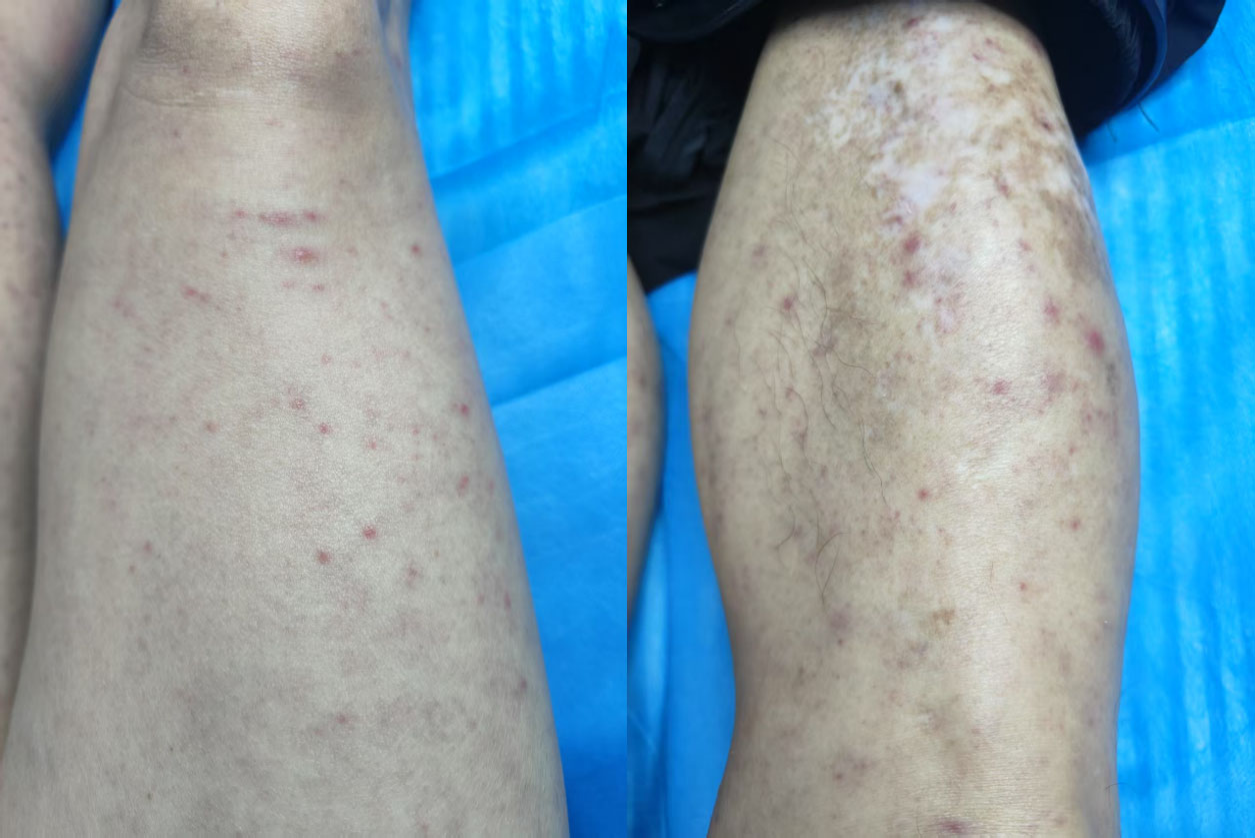
Clinical presentation of cutaneous lesions on the lower extremities. Multiple erythematous nodular lesions with well-defined borders and mild tenderness were observed, predominantly on the bilateral lower legs.

However, on day 50 post-infusion (June 27, 2025), the patient developed multiple erythematous nodular lesions on the trunk and lower extremities, predominantly on the bilateral lower legs. The lesions were round to oval, measuring 1–2 cm in diameter, with well-defined borders and mild tenderness (**Figure 1**). Notably, the patient had no fever, arthralgia, or systemic symptoms, and the rash showed no signs of rapid progression or necrosis. Laboratory evaluation revealed a white blood cell count of 2.27 × 10^9^/L, absolute neutrophil count of 1.43 × 10^9^/L, and C-reactive protein of 0.68 mg/dL.

**Figure 2:**
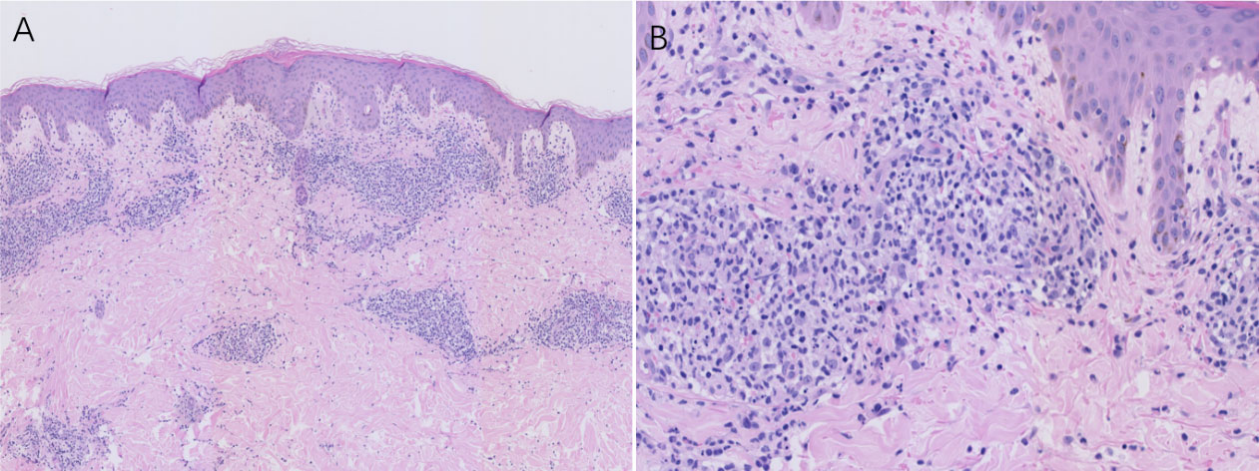
Histopathological findings of skin biopsy. **(A)** Low-power view (H&E, magnification ×100) showing a dense, wedge-shaped dermal inflammatory infiltrate. **(B)** High-power view (H&E, magnification ×400) revealing a predominance of mature neutrophils, along with histiocytes and significant leukocytoclasia (nuclear dust). Papillary dermal edema is also noted. No features of true vasculitis are identified.

A punch biopsy of a lower extremity lesion was performed on June 30, 2025. The rash resolved spontaneously after approximately 10 days in early July without glucocorticoid or other immunosuppressive intervention. Histopathological examination (**Figure 2**) showed largely normal epidermis, dermal papillary edema, and dense perivascular and interstitial infiltration of neutrophils, histiocytes, and nuclear dust distributed in a wedge-shaped pattern throughout the full thickness of the dermis. No definitive evidence of vasculitis was observed.

**Figure 3:**
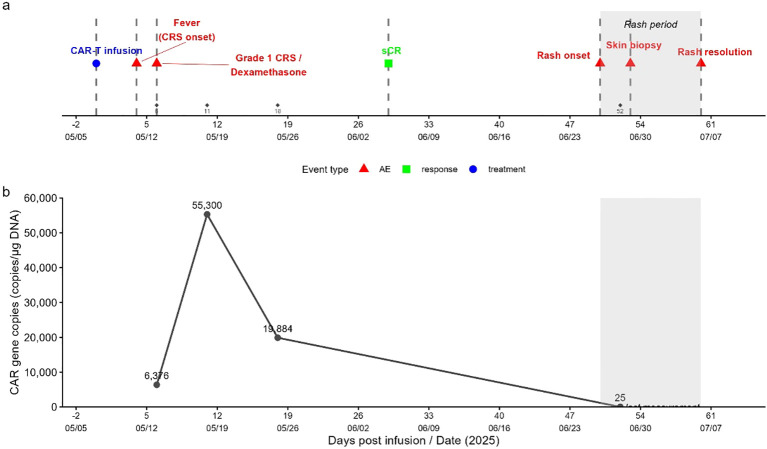
Clinical timeline and CAR-T cell expansion. **(A)** Timeline of clinical events after BCMA CAR-T infusion. Grey dashed lines mark event days; shapes and colors indicate event type (treatment, adverse event, response). The grey shaded area (days 50–60) represents the rash period. Bottom grey diamonds show days when CAR copy numbers were measured. **(B)** CAR gene copy numbers at four time points (days 6, 11, 18, 52). Points are connected by straight lines which are drawn only for visual trend assessment and do not imply continuous monitoring. The y-axis is linear with 10,000-unit intervals. The last measurement (day 52, 25 copies/μg DNA) fell within the rash period. X-axis shows days post-infusion and calendar date (May–July 2025) with 7-day ticks.

These histopathological findings are characteristic of Sweet syndrome. However, given the absence of fever and leukocytosis, and the self-limited course, the diagnosis did not fully meet the classical diagnostic criteria. In our differential diagnosis, erythema multiforme was considered unlikely because of the absence of target lesions, epidermal necrosis, and interface changes histologically. Erythema nodosum was largely excluded by the lack of tender subcutaneous nodules and the absence of septal panniculitis on biopsy. Pyoderma gangrenosum was considered improbable given the non-ulcerative, non-pustular appearance and the self-limited course without pathergy. Leukocytoclastic vasculitis was ruled out by the absence of fibrinoid necrosis of vessel walls and the lack of vasculitis on histopathology. An infectious etiology was judged unlikely due to the negative systemic infection workup, the absence of tissue necrosis or suppuration, and the spontaneous resolution without antimicrobial therapy. A simple drug eruption was also considered but was felt to be less consistent with the dense neutrophilic infiltrate and the relatively long interval since the last administration of any potential culprit medication. After excluding other differential diagnoses including erythema multiforme, erythema nodosum, pyoderma gangrenosum, and leukocytoclastic vasculitis, a final diagnosis of BCMA CAR-T therapy-associated “Sweet-like syndrome” was established. The patient has been followed for approximately 4 months after the rash resolved, and during this period the cutaneous lesions did not recur, and the patient has maintained stringent complete remission for MM. The timeline of events and CAR-T cell copy-number kinetics are presented in **Figure 3**.

## Discussion

Sweet syndrome was first described by Sweet in 1964 as an acute febrile neutrophilic dermatosis characterized by fever, peripheral leukocytosis, and dense dermal neutrophilic infiltration (10). The cutaneous lesions in our case, occurring 50 days after BCMA CAR-T infusion, exhibited the histopathological hallmarks of Sweet syndrome: dense dermal neutrophilic infiltration, nuclear dust, and absence of vasculitis. However, the patient lacked the classical clinical features of fever, arthralgia, and peripheral neutrophilia. According to the von den Driesch diagnostic criteria (11), this case does not fully meet the requirements for classical Sweet syndrome but rather aligns with “Sweet-like syndrome,” which has been increasingly reported in the context of specific drug triggers (12, 13).

Several lines of evidence suggest a possible causal association between this dermatologic event and CAR-T therapy. First, the temporal relationship is compelling: the rash developed on day 50 post-infusion, during the period when the peripheral CAR-T cell copy numbers maintained stable levels and were exerting antitumor effects. Second, alternative etiologies were carefully excluded. Although granulocyte colony-stimulating factor (G-CSF) (14) and multiple myeloma itself (15) have been reported to induce Sweet syndrome. G-CSF was administered only briefly during the CRS period, well before the onset of rash, and the patient’s sCR status with minimal residual disease negativity effectively excludes paraneoplastic mechanisms. No other potential culprit medications (e.g., lenalidomide, bortezomib) had been administered for over three months prior to rash development. Notably, Kaffenberger and colleagues reported a case of pyoderma gangrenosum, another neutrophilic dermatosis within the same disease spectrum, following CAR-T therapy for diffuse large B-cell lymphoma (16). That further suggests that CAR-T therapy may predispose to such conditions. Nonetheless, without detection of CAR transgene or CAR-specific immune activity within the skin lesion, the relationship remains circumstantial.

The mechanism underlying this cutaneous event warrants discussion. BCMA expression is almost exclusively restricted to plasma cells and plasmablasts, with no significant expression in normal skin tissues (17). Therefore, the Sweet-like lesions observed in this patient cannot be explained by classical “on-target/off-tumor” toxicity, in contrast to the cutaneous adverse events associated with GPRC5D-targeted immunotherapies (such as talquetamab or GPRC5D CAR-T), where rashes, desquamation, and onycholysis result from direct targeting of GPRC5D physiologically expressed in keratinized skin tissues (18). This distinction suggests that BCMA CAR-T therapy likely induces cutaneous inflammation through indirect mechanisms.

We hypothesize that CAR-T cell expansion and tumor cell killing release substantial quantities of proinflammatory cytokines, including IFN-γ and IL-6 (19). This cytokine release not only triggers CRS but may also systemically activate circulating neutrophils, priming them for enhanced responsiveness. Subsequently, these primed neutrophils may be recruited to the skin by local chemotactic signals potentially released by activated keratinocytes, resulting in dermal infiltration (20). This mechanism can be conceptualized as a “functional” cutaneous response, wherein the antitumor effects of CAR-T cells reshape systemic immune status, leading to a bystander effect in distal sites such as the skin. This model remains hypothetical, as no local cytokine profiling or immunophenotyping of the skin infiltrate was performed.

The self-limited course of the cutaneous lesions in this case aligns with prior reports indicating that most CAR-T-related cutaneous toxicities are grade 1–2 and resolve spontaneously (4, 21). Notably, the rash occurred while the patient was in sCR and peripheral CAR-T cell copy numbers remained stable (approximately 25 copies/μg DNA), rather than during the acute CRS phase. This observation raises the possibility that such delayed, self-limited skin reactions may represent a “byproduct” of sustained, effective immune surveillance mediated by CAR-T cells. Whether this reflects a controlled immune reconstitution phenomenon or merely self-limited local inflammation requires further investigation.

From a clinical perspective, this case offers important lessons. The immunologic effects of CAR-T therapy extend temporally beyond the acute CRS phase; delayed, tissue-specific inflammation may occur even after resolution of systemic cytokine release. For atypical rashes lacking fever or leukocytosis, a low threshold for skin biopsy is essential to avoid misdiagnosis as simple drug eruption and to prevent unnecessary broad-spectrum antibiotics or high-dose corticosteroids driven by concerns for severe infection or atypical CRS. The self-limited nature of this case also suggests that for grade 1–2 cutaneous toxicities without rapid progression or systemic symptoms, close observation rather than immediate systemic immunosuppression may be appropriate, provided a pathological diagnosis has been confirmed. Of course, rapidly progressive, ulcerative, or necrotic grade 3–4 skin reactions warrant prompt intervention.

Several limitations should be acknowledged. As a single case report with retrospective observation, the proposed mechanisms remain speculative and require validation through further basic research, including single-cell sequencing or spatial transcriptomics of lesional tissue. Additionally, we were unable to analyze the local cytokine profile at the peak of rash development or perform CAR gene detection on infiltrating T cells to determine whether CAR-T cells directly homed to the skin. Future studies with larger case series and prospective registries are needed to clarify the incidence, risk factors, and prognostic implications of such rare events.

## Conclusion

This case represents, to our knowledge, the first report of Sweet-like syndrome after BCMA CAR-T therapy, thereby expanding the recognized spectrum of immune-related adverse events associated with this treatment. The delayed onset, characteristic histopathology, and spontaneous resolution may reflect an indirect, immune-mediated process associated with sustained CAR-T cell activity. Our experience underscores the importance of maintaining a low threshold for skin biopsy in patients who develop atypical, delayed-onset rashes post-CAR-T. For low-grade presentations without systemic features, tissue diagnosis can enable a conservative management approach and help avoid unnecessary empiric corticosteroid therapy.

## Data Availability

The original contributions presented in the study are included in the article/supplementary material. Further inquiries can be directed to the corresponding authors.
